# Nitazene opioids and the heart: Identification of a cardiac ion channel target for illicit nitazene opioids

**DOI:** 10.1016/j.jmccpl.2024.100118

**Published:** 2024-12

**Authors:** Jules C. Hancox, Yibo Wang, Caroline S. Copeland, Henggui Zhang, Stephen C. Harmer, Graeme Henderson

**Affiliations:** a School of Physiology, Pharmacology and Neuroscience, Biomedical Sciences Building, University Walk, Bristol BS8 1TD, UK; b Biological Physics Group, Department of Physics and Astronomy, The University of Manchester, M13 9PL, UK; c Centre for Pharmaceutical Medicine Research, Institute of Pharmaceutical Science, King's College London, 150 Stamford Street, London SE1 9NH, UK; d National Programme on Substance Use Mortality, London, UK

**Keywords:** hERG, Etonitazene, Isotonitazene, Long QT, Metonitazene, Nitazene, Opioid, Protonitazene, QT interval, Torsades de pointes

## Abstract

The growing use of nitazene synthetic opioids heralds a new phase of the opioid crisis. However, limited information exists on the toxic effects of these drugs, aside from a propensity for respiratory depression. With restricted research availability of nitazenes, we used machine-learning-based tools to evaluate five nitazene compounds' interaction potential with the hERG potassium channel, a key drug antitarget in the heart. All nitazenes were predicted to inhibit hERG with low μM IC_50_ values. These findings indicate a potential for proarrhythmic hERG block by nitazene opioids, warranting detailed cardiac safety evaluations of these drugs.

## Graphical abstract

**Figure:**
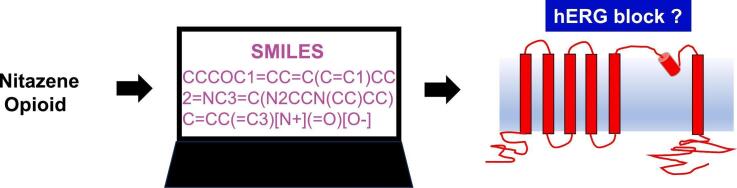

## 1 Introduction

Nitazenes are synthetic opioids from the benzimidazole family that were first developed in the pharmaceutical industry in the 1950s [1]. They preferentially target μ (mu) opioid receptors, with some nitazenes exhibiting higher affinity than fentanyl [2]. They are many times more potent than heroin [3] and have been used in preclinical addiction research [1,4]. In early human trials of some nitazenes, significant respiratory depression was seen and nitazenes have never been approved for medical use in humans [1,4].

Due to their strong psychotropic effects, opioids have long been drugs of illicit use. Fentanyl and other synthetic opioids were involved in 80,411 of 106,699 (~75 %) drug-involved overdose deaths in the USA in 2021. 2-benzylbenzimidazole nitazenes include etonitazene, isotonitazene, metonitazene and protonitazene [1,4] while the related benzimidazolene brorphine is also subject to illicit use. The first nitazene drug to be identified as a street drug in Europe in 2019 was isotonitazene (aka “Iso” or “Tony”) [4], which is an analogue of etonitazene [1,4]. In cases of toxicity or fatality in the USA, isotonitazene predominated from 2019 until it became scheduled, with the pattern then shifting to metonitazene [5]. In 2023 brorphine, isotonitazene, metonitazene and protonitazene were detected in wastewater in Australia [6], while in 2024 protonitazene was detected for the first time in wastewater from 2 states in the USA [7]. This is indicative of community use of these drugs. Reported nitazene-linked deaths may only be the tip of the iceberg as emerging drugs are not routinely tested for and tests are still in development [3].

The dominant health risk in nitazene use arises from respiratory depression/arrest [1,4]. However, given that illicit nitazene use is a recently emerging problem, there is a paucity of pharmacokinetic and pharmacodynamic information on these drugs [4] and it is possible that other secondary unwanted effects of nitazenes may be overlooked. Metonitazene overdose has been linked to cardiac arrest [8]. A preclinical study of etazene (etodesnitazene) performed on *Danio rerio* has reported dose dependent developmental toxicity including cardiotoxicity, evidenced by heart malformation and bradycardia in embryos [9]. Exposure to high etazene concentrations produced arrhythmia/heart block in this model [9].

Recent reviews of opioids and arrhythmia have concluded that methadone has potential for QT prolongation and Torsades de Pointes (TdP) at low concentrations, while tramadol and oxycodone have intermediate risk and may prolong the QT interval and cause TdP at high doses [10,11]. Similar to other QT prolonging drugs, methadone and oxycodone, as well as fentanyl, can inhibit hERG channels [[12], [13], [14]]. There are no comparable data currently available for nitazenes. The ‘gold standard’ method for interrogating whether drugs can inhibit hERG is patch-clamp evaluation of hERG channel inhibition *in vitro*. For compounds with restricted availability, *in silico* screens exist that can be employed early in the evaluation process; we have recently employed this approach to demonstrate hERG-blocking propensity of a number of illicitly used synthetic cannabinoid receptor agonists (SCRAs) [15]. Here we employed four publicly accessible online computational tools to evaluate five nitazene compounds.

## 2 Methods

hERGSPred utilised machine-learning-based models trained on 12,850 compounds to produce a consensus model with an accuracy of 0.839 [16]. PredhERG was originally developed from a dataset of 5984 compounds [17]. We employed these models in our recent evaluation of SCRA compounds [15]. PredhERG 5.0 is a recent PredhERG update built on an integrated database of 14,364 compounds. It offers binary (blocker/non-blocker), multiclass (non-blocker, weak-moderate or strong blocker) and regression predictions. Significantly, the multiclass prediction includes potency (pIC_50_) predictions, so we focused on this aspect of the PredhERG 5.0 model for the present study. ADMETSar is a long-standing tool for prediction of ADMET properties, giving a probability and verbal descriptor of hERG inhibition strength [18] ADMETSar 2.0 is a 2019 update of ADMETSar built on further training data and with more ADMET predictions [19]. PredhERG and ADMETSar 2 include whether or not a chemical structure falls within the applicability domain (AD) of the model. All nitazenes fell within the ADs of both models. All tools have web-based user interfaces and when this study was conducted (between March and May 2024) were freely available for online use. SMILES structures of etonitazene, isotonitazene, metonitazene and protonitazene) were entered into each tool. We previously used E-4031 and escitalopram as positive controls and atenolol was included as a non-hERG blocker negative control [15]. For the present study, fentanyl was used as a positive control for hERG potency prediction with the PredhERG 5.0 multiclass model. Ventricular action potential (AP) simulations were performed with the open source “AP Predict” online cardiac electrophysiology simulator, at 0.5, 1 and 2 Hz. Effects of protonitazene on human atrial cell APs were simulated by using the Colman et al. model [20] at rates of 0.5 Hz, 1 Hz and 2 Hz. To evaluate the effects of protonitazene on the electrical excitation of and conduction through ventricular tissue, the O'Hara et al. [21] human ventricle model was used, and a 1D transmural strand model was constructed that considered the intrinsic electrical heterogeneity of epi-, mid- and *endo*-layers of myocardium [22]. Effects of protonitazene on APs in the 1D strand and conduction velocity and vulnerability of cardiac tissue to uni-directional conduction block in response to premature stimuli were quantified using methods described in [22].

## 3 Results and discussion

**Fig. 1:**
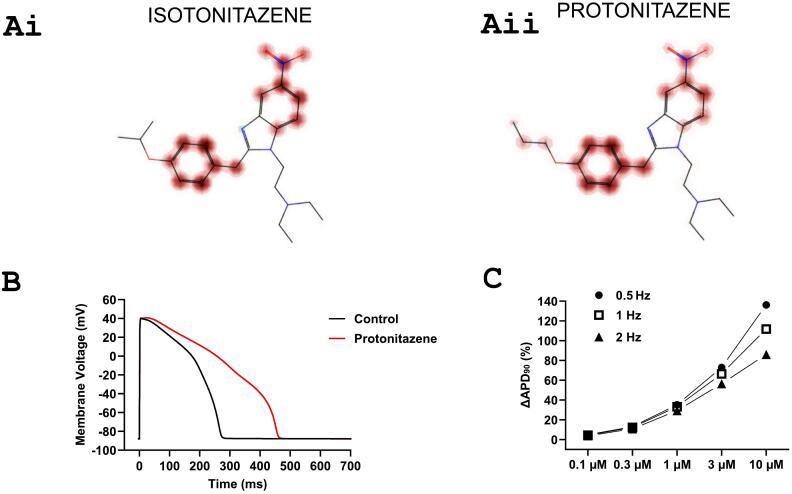
*Drug structures of isotonitazene and protonitazene and illustrative prediction of action potential (AP) prolongation for simulated hERG block by protonitazene.* **(A)** Isotonitazene **(Ai)** and protonitazene **(Aii)** are both 6-nitro-2 benzylbenzimidazole members of the benzimidazole group of synthetic opioids. The PredhERG multiclass prediction that yielded predicted IC_50_ values highlighted (in magenta) parts of each molecule predicted to contribute to hERG channel inhibition by these compounds. For both drugs, aromatic rings (including that in the benzimidazole moiety) were highlighted as likely contributors to hERG interaction. **(B,C)** Ventricular AP simulations were conducted in April 2024 using the “AP Predict” online cardiac electrophysiology simulator [18]. The O'Hara Rudy CiPA ventricular AP model was selected and a pIC_50_ value for I_Kr_ of 5.904 (corresponding to an IC_50_ of 1.25 μM) was entered (based on PredhERG protonitazene evaluation in Table 1). APs were elicited at 0.5, 1 and 2 Hz. (**B**) shows APs in control and at a concentration of 3.41 μM (at 1 Hz). (**C**) Extent of prolongation of APD_90_ (ΔAPD_90_ (%)) plotted against 5 protonitazene concentrations (0.1, 0.3, 1, 3 and 10 μM) at 3 frequencies (0.5, 1 and 2 Hz). The observed ΔAPD_90_ (%) values ranged from 4.38 to 111.77 % at 1 Hz. These simulations assume selective drug activity against hERG/I_Kr_. (For interpretation of the references to colour in this figure legend, the reader is referred to the web version of this article.)

Table 1 shows the prediction outcomes for the five nitazenes assessed in this study. hERGSPred and ADMETSar 2.0 rated all compounds as having a high probability of being hERG inhibitors (the probability was 0.99 for all compounds with hERGSPred and ranged between 0.8346 and 0.8939 for ADMETSar 2.0). ADMETSar 1 rated etonitazene, metonitazene, isotonitazene and protonitazene to be strong hERG inhibitors (with probabilities ranging between 0.8210 and 0.8698) and brorphine also to be a strong hERG inhibitor, albeit with a lower probability of 0.5556). All compounds were within the applicability domain of PredhERG 5.0 and with binary prediction all except etonitazene were classified as hERG blockers (Table 1). Multiclass evaluation by PredhERG 5.0 classified all 5 compounds as moderate blockers with estimated IC_50_ values of between 1.25 and 1.84 μM for etonitazene, metonitazene, isotonitazene and protonitazene and of 4.71 μM for brorphine. Fig. 1A shows visualized outputs of multiclass evaluation of isotonitazene (Fig. 1Ai) and protonitazene (Fig. 1Aii), highlighting in burgundy parts of these molecules considered likely to contribute to interactions with hERG. For comparison with nitazenes, PredhERG multiclass analysis evaluated the opioid fentanyl as having an IC_50_ of 1.76 μM, which is in good agreement with experimentally obtained IC_50_ values for fentanyl of 0.8–1.8 μM [12,23].

**Table 1:** Predicted hERG channel inhibition for five nitazene drugs. SMILES structures were input into “PredhERG”, “hERGSPred” ADMETSar and ADMETSar 2.0 web-based prediction models between March and April 2024. Links to PubChem information, including canonical SMILES, are included below each drug name.

Compound	PredhERG	ADMETSar 1 (Type Probability)	ADMETSar 2 (Y/N Probability)	hERGSPred (Y/N Probability)
Etonitazene (PubChem entry)	Binary: Nonblocker (51 %) Multiclass: Moderate blocker: IC_50_ 1.80 μM	Strong 0.8472	Y 0.8939	Y 0.99
Isotonitazene (PubChem entry)	Binary: Blocker (68 %) Multiclass: Moderate blocker: IC_50_ 1.84 μM	Strong 0.8210	Y 0.8784	Y 0.99
Metonitazene (PubChem entry)	Binary: Blocker (54 %) Multiclass: Moderate blocker: IC_50_ 1.50 μM	Strong 0.8698	Y 0.8889	Y 0.99
Protonitazene (PubChem entry)	Binary: Blocker (68 %) Multiclass: Moderate blocker: IC_50_ 1.25 μM	Strong 0.8546	Y 0.8886	Y 0.99
Brorphine (PubChem entry)	Binary: Blocker (54 %) Multiclass: Moderate blocker: IC_50_ 4.71 μM	Strong 0.5556	Y 0.8346	Y 0.99

**Fig. 2:**
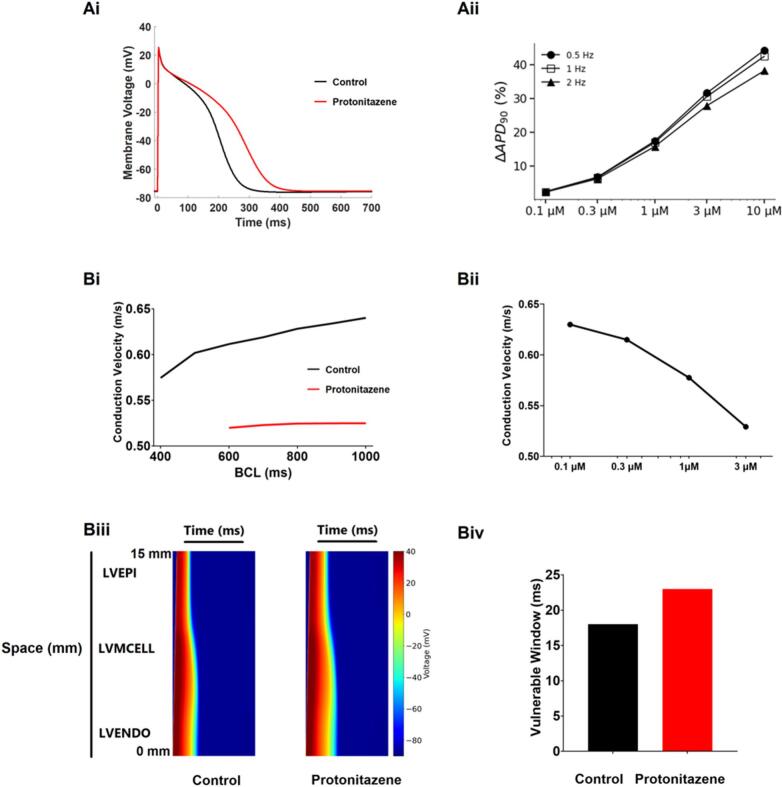
*Effects of I*_*Kr*_*inhibition by protonitazene on human atrial APs and ventricular tissue excitation.* (**Ai**) Simulated human atrial APs using the Colman et al. [21] model in control and with 3.41 μM protonitazene (the drug's potency against I_Kr_ was the same as for Fig. 1). APs were elicited by suprathreshold stimuli at a basic cycle length of 1000 ms (1 Hz) (BCL = 1000 ms). (**Aii**) ΔAPD_90_ (%) for atrial APs plotted against 5 protonitazene concentrations (0.1, 0.3, 1, 3 and 10 μM) at 3 frequencies (0.5, 1 and 2 Hz). (**Bi,Bii**) Frequency- (Bi) and concentration- (Bii) dependent effects of protonitazene on the conduction velocity computed from the ventricular strand model. Concentration used for Bi was 3.41 μM and stimulation frequency for Bii was 1 Hz. Note Y (CV) axis starts at 0.5 m/s not 0. (**Biii-Biv)** Illustration of effects of 1 μM protonitazene on the propagation of excitation waves (Biii) and inducibility (Biv) of uni-directional conduction block in response to a premature stimulus. The inducibility was measured by the width of the VW at the ENDO-MID junction of the 1D strand model [22]. The 1D strand model had a length of 15 mm, which was discretised by a spatial resolution of 0.15 mm, forming 100 nodes of cells.

There are no desirable clinical plasma concentration ranges of nitazenes. In one protonitazene overdose-linked fatality, the concentration of protonitazene measured in cardiac blood was 1400 ng/ml (equivalent to 3.41 μM; it was not specified whether this represented total or free/unbound protonitazene) [24]. For illustrative purposes, we have used this information together with the hERG IC_50_ of 1.25 μM provided by PredhERG (Table 1) in human ventricular AP simulations (Fig. 1B,C). Fig. 1B shows overlaid APs in control and with 3.41 μM protonitazene. The AP duration at 90 % repolarization (APD_90_) was prolonged by 70.9 %. Fig. 1C shows that protonitazene prolonged APD_90_ over a concentration range between 0.1 and 10 μM. APD_90_ prolongation was greater at a slower stimulation rate (0.5 Hz) and was smaller at a faster stimulation rate (2 Hz). The differences in extent of APD_90_ prolongation were small for low concentrations (APD_90_ prolongation by 4.77 %, 4.38 %, and 3.80 % at 0.5 Hz, 1 Hz and 2 Hz respectively, at 0.1 μM) and was particularly evident at higher concentrations (APD_90_ prolongation by 136.17 %, 111.77 %, and 86.09 % at 0.5 Hz, 1 Hz and 2 Hz respectively, at 10 μM). Qualitatively similar results were seen in atrial cell AP simulations (Fig. 2A), in which clear prolongation of APD_90_ (Fig. 2Ai,Aii) was seen that was greater at lower than higher stimulation frequencies (particularly at higher concentrations). Notably, in both ventricular and atrial simulations some APD prolongation is anticipated to occur at sub-micromolar drug concentrations (assuming selective hERG/I_Kr_ blockade).

Conduction through a 1D transmural ventricular strand was evaluated to probe potential nitazene effects at the tissue level. Fig. 2Bi shows that simulated application of 3.41 μM protonitazene decreased conduction velocity (CV) over a range of cycle lengths, while Fig. 2Bii shows concentration dependent reduction of CV at a BCL of 1000 ms (1 Hz). Fig. 2Biii shows the conduction of excitation waves in a 1D transmural ventricular strand in response to a stimulus applied to the ENDO-end of the 1D strand model for both control (left panel) and protonitazene (concentration of 1 μM; right panel) conditions. Fig. 2Biv shows measurements of the vulnerable window (VW) to unidirectional block induced by a second stimulus applied to the ENDO-MID junction after an initiation excitation: the VW increased in the simulated presence of protonitazene, consistent with a greater susceptibility to re-entry in the presence than absence of the drug.

It is significant that many nitazene users are polydrug users [5,24] and that nitazenes can be found mixed with other drugs [4]. Thus, even modest hERG block by nitazene drugs could be of importance when a nitazene drug is combined with other agents that can inhibit the hERG channel, as is the case for many psychotropic drugs. While there was good agreement across the *in silico* tools used to evaluate nitazenes in this study, their predictions require now to be evaluated through *in vitro* electrophysiological studies of recombinant hERG channels. A further limitation of the present study is that it focuses on hERG alone and we cannot rule out that nitazenes may interact with other cardiac ion channels or electrogenic transporters. For example, the structurally distinct opioid methadone has been demonstrated to inhibit cardiac inward rectifier K^+^ current [25], Na_v_1.5 Na^+^ channels [26] and SK2 K^+^ channels [27]. Thus, effects of nitazenes on other cardiac ionic currents than I_Kr_/hERG should also be investigated in future work. Although it is important to highlight these potential limitations, they do not fundamentally affect the principal conclusion of our study: our findings identify a potential cardiac target for nitazene drugs. Certainly, it would be prudent for people who use nitazenes – or other illicit substances that have been found to be contaminated with nitazenes (*e.g.*, counterfeit diazepam and heroin tablets) – to adopt caution in the use of other illicit or licensed drugs that have a known association with drug-induced long QT syndrome. Given the growing problem of nitazene use, the results of this study highlight an imperative for further investigation of effects of these illicit drugs on cardiac electrophysiology.

## Declaration of Generative AI and AI assisted technologies in the writing process.

None

## CRediT authorship contribution statement

**Jules C. Hancox:** Writing – original draft, Methodology, Investigation, Funding acquisition, Formal analysis, Data curation, Conceptualization. **Yibo Wang:** Data curation, Formal analysis, Investigation, Writing – review & editing. **Caroline S. Copeland:** Writing – review & editing, Investigation. **Henggui Zhang:** Data curation, Formal analysis, Methodology, Writing – original draft, Writing – review & editing. **Stephen C. Harmer:** Writing – review & editing, Investigation, Funding acquisition. **Graeme Henderson:** Writing – review & editing, Investigation, Funding acquisition.

## Declaration of competing interest

The authors declare that they have no known competing financial interests or personal relationships that could have appeared to influence the work reported in this paper.
